# Optically Controlling Broadband Terahertz Modulator Based on Layer-Dependent PtSe_2_ Nanofilms

**DOI:** 10.3390/nano13050795

**Published:** 2023-02-21

**Authors:** Hong Su, Zesong Zheng, Zhisheng Yu, Shiping Feng, Huiting Lan, Shixing Wang, Min Zhang, Ling Li, Huawei Liang

**Affiliations:** 1 Key Laboratory of Optoelectronic Devices and Systems of Ministry of Education and Guangdong Province, Shenzhen University, Shenzhen 518060, China; 2 Shenzhen Key Laboratory of Laser Engineering, College of Physics and Optoelectronic Engineering, Shenzhen University, Shenzhen 518060, China

**Keywords:** terahertz, modulator, photoconductivity, PtSe_2_

## Abstract

In this paper, we propose an optically controlling broadband terahertz modulator of a layer-dependent PtSe_2_ nanofilm based on a high-resistance silicon substrate. Through optical pump and terahertz probe system, the results show that compared with 6-, 10-, and 20-layer films, a 3-layer PtSe_2_ nanofilm has better surface photoconductivity in the terahertz band and has a higher plasma frequency *ω_p_* of 0.23 THz and a lower scattering time *τ_s_* of 70 fs by Drude–Smith fitting. By the terahertz time-domain spectroscopy system, the broadband amplitude modulation of a 3-layer PtSe_2_ film in the range of 0.1–1.6 THz was obtained, and the modulation depth reached 50.9% at a pump density of 2.5 W/cm^2^. This work proves that PtSe_2_ nanofilm devices are suitable for terahertz modulators.

## 1. Introduction

Being the transition zone between electronics and photonics, terahertz (THz) waves have unique and fascinating properties that attract extensive research. It has been applied in a variety of fields such as security [1], biological nondestructive testing [2], and communication [3,4]. At the same time, terahertz devices such as modulators [5,6], absorbers [7,8], detectors [9], and so on have also been developed. Among them, the terahertz modulator, an active device for controlling terahertz propagation, is an important device in the field of 6G communication [10] and has become a research trend.

Different types of modulators have been reported in previous studies, mainly based on structures and materials. On the one hand, the structures designed for terahertz modulators are photonic crystals [11,12,13], artificial electromagnetic materials [14,15], ultrasurface microstructures [16,17], and so on. This kind of the device is characterized by accurate design and high-precision etching; but it also has the limitations of having a narrow working band, high processing accuracy, and limited optional materials, and once formed, it cannot be changed. On the other hand, the materials for terahertz modulators can be selected from organic materials [18,19], semiconductors [20,21], polymers [22], and two-dimensional materials [5,6,23,24]. Among them, the application of two-dimensional materials in terahertz modulators is an emerging research hotspot, which has rapidly developed in recent years. Two-dimensional material modulators can use not only their own properties, such as anisotropy and near-zero band gap, to carry out passive modulation but also active field to realize the active modulation of devices [15,25]. Moreover, optical controlling is more widely used in terahertz modulators than electrical controlling because of its faster modulation speed.

One needs to find the material with high carrier mobility, short carrier relaxation time, and significant surface conductivity for the modulator [26,27]. As novel transition metal dichalcogenide (TMDCs) materials, PtSe_2_ and its parental compounds such as PtTe_2_, PdTe_2_, and NiTe_2_ have been widely studied by researchers for their unique energy band structure and novel physical properties [28,29,30,31]. However, PtSe_2_ stands out due to its unique properties of having high carrier mobility, an adjustable band gap of 0–1.2 eV, and fast response characteristics [32,33,34]. Although it has been demonstrated that PtSe_2_ films can be used as high-efficiency terahertz modulators through the properties of the materials as mentioned by Alka Jakhar [35], how the photoconductivity properties of PtSe_2_ films affects the modulation depth still has not been verified.

In this paper, an optically controlling broadband terahertz modulator of layer-dependent PtSe_2_ nanofilms based on a high-resistance silicon substrate is proposed. By the terahertz time-domain spectroscopy (THz-TDS) system and optical pump and terahertz probe (OPTP) experimental setup, the optical modulation and photoconductivity characteristics of different PtSe_2_ films, respectively, are investigated.

## 2. Sample Characterization

PtSe_2_ films with different layers of 3L, 6L, 10L, and 20L were grown on a sapphire substrate by chemical vapor deposition (CVD) and then transferred to a high-resistance silicon substrate (resistivity > 10,000 Ω cm, 450 μm thickness) with a size of about 1cm×1cm for the study of optical modulation characteristics. On the other hand, the samples used in the OPTP experiment, Raman spectra, and UV–visible spectra for studying the photoconductivity characteristics and characterization of different layers of PtSe_2_ films are based on a sapphire substrate. When we measured the band gap of PtSe_2_ films, another sapphire substrate without a PtSe_2_ film is required as a control to minimize its influence.

**Figure 1:**
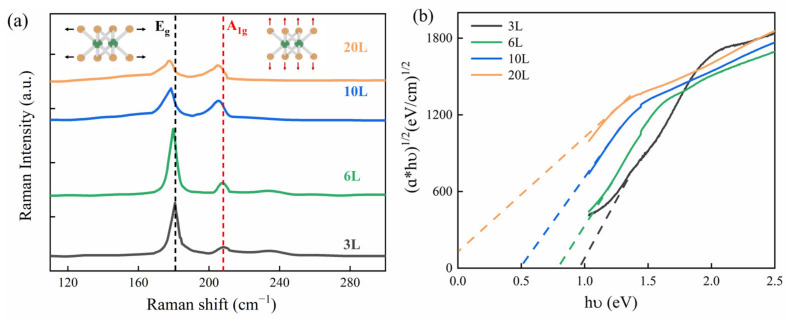
(**a**) Raman spectra of PtSe_2_ films with different layers; in the illustration, the yellow circle is Se atom, the green circle is Pt atom, and the arrows indicate the direction of atoms’ motion. (**b**) Relationship between absorption coefficient and band gap of PtSe_2_ films with different layers.

By using a Horiba Jobin Yvon LabRAM HR Evolution spectrometer, the Raman spectra of PtSe_2_ films with different layers are shown in Figure 1a. The two Raman peaks E_g_ and A_1g_ resulting from intralayer in-plane vibration and out-of-plane vibration of Se atoms are observed at near 180 cm^−1^ and 208 cm^−1^, respectively, which is consistent with previous research results [36,37]. One can also see that the peak positions of both E_g_ and A_1g_ modes have a certain red shift as the layer numbers increase, and the intensity of the E_g_ mode is higher than that of the A_1g_ mode. It indicates that the long-distance Coulomb interaction dominates the atomic vibration and structure changes caused by the superposition of PtSe_2_ films [32]. In addition, the absorption spectra of the PtSe_2_ films were measured by a UV–visible spectrophotometer. The relationship between the absorption coefficient and band gap of PtSe_2_ films with 3L, 6L, 10L, and 20L can be obtained by the Tauc plot method as shown in Figure 1b, and the corresponding band gaps of PtSe_2_ films with a different layer are 0.98 eV, 0.78 eV, 0.48 eV, and 0 eV, respectively. It shows that the 20L PtSe_2_ film is a semimetal, just like a bulk material, which is in good agreement with previous reports [32,36,38]. Therefore, in order to explore the modulation performance of this thickness-dependent material, we need to study further its surface conductivity characteristics.

## 3. Results and Discussion

**Figure 2:**
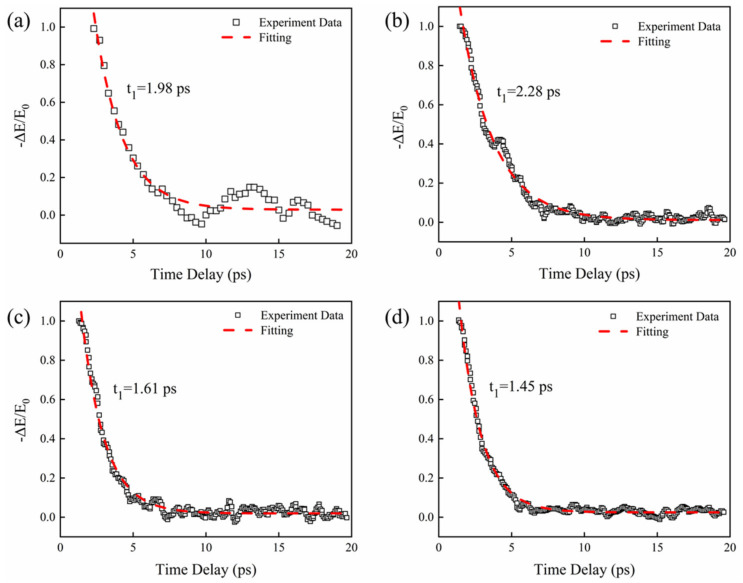
Normalized terahertz transmission change (−Δ*E*/*E_0_*) induced by the pump power of 150 mW in (**a**) 3L PtSe_2_ film; (**b**) 6L PtSe_2_ film; (**c**) 10L PtSe_2_ film; and (**d**) 20L PtSe_2_ film.

The OPTP and THz-TDS systems in Ref. [36] were used to measure the carrier dynamics of the sample. In order to achieve the optimal modulation performance, the pump laser is obliquely incident to the surface of the material, and the pump spot is larger than the terahertz spot. In the OPTP system, the spot diameter of the terahertz beam is about 8 mm, and the diameter of the pump beam for the femtosecond lasers is about 1 cm. By the OPTP system, the peak electric field intensity of the transmission terahertz wave *E*_0_ for the PtSe_2_ film is obtained without excitation light. Furthermore, there exists a change of the transmission terahertz peak electric field intensity Δ*E* when the optical wave excites the sample, where Δ*E* = *E* − *E*_0_, with *E* representing the peak electric field intensity of the terahertz wave with excitation light. The relative change of *−*Δ*E/E_0_* with the detection time delay is measured for PtSe_2_ films with different layers, as shown in Figure 2. One can obtain that the relaxation times of 3L, 6L, 10L, and 20L PtSe_2_ films at a pump power of 150 mW are 1.98 ± 0.14 ps, 2.28 ± 0.03 ps, 1.61 ± 0.02 ps, and 1.45 ± 0.02 ps, respectively. The relaxation time of carriers in multilayer materials is short. Because the density of defect states in materials increases with the increase in film thickness, the excitons are more easily captured by the defect states of materials. Thus, the recombination velocity of the carriers is increased. However, the thinner the PtSe_2_ films, the longer the relaxation time. This is because the defect state density of a few-layer PtSe_2_ film is small, which is not enough to capture all the photogenerated excitons [39]. In addition, the remaining excitons in the conduction band relax to the bottom of the conduction band at a faster recombination time constant in a few-layer PtSe_2_ film.

In Figure 2, the normalized relative field intensity change of *−*Δ*E/E_0_* for different PtSe_2_ films induced by the same pump density manifests a single exponential decay. It can be seen that the relative field intensity change with the detection time delay shows a rapid increase and follows by attenuation. This is due to the fact that after the femtosecond laser pulse is incident on the sample, the photogenerated electrons and holes can firstly generate excitons. Then, the excitons are captured by the defect states in the material or recombined in the form of Auger exciton–exciton annihilation. This process typically takes a few picoseconds to generate. Therefore, the relaxation process can be fitted by the convolution of a single exponential function. The formula is given as follows [40]:

(1) $$-\frac{\Delta E}{E_{0}}=A_{1}\exp\left(-\frac{t}{t_{1}}\right)$$

where *A*_1_ is a constant, *t* is the delay time of excitation light, and *t*_1_ is the carrier relaxation time.

The conductivity Δ*σ*(*ω*) is extracted from the measured Δ*E*/*E*_0_ data by changing the delay time *t* between the pump and terahertz probe waves. The change of conductivity with frequency is shown as [41]

(2) $$\Delta \sigma \left(\omega \right)=-\frac{1+n_{s}}{Z_{0}}\frac{\Delta E_{t}\left(\omega \right)}{E\left(\omega \right)}$$

where *n_s_* is the substrate refractive index, *Z*_0_ is the free space impedance, and Δ*E_t_*(*ω*) = *E_t_*(ω) − *E*(ω), with *E_t_*(ω) and *E*(ω) being the transmission spectral amplitude of the sample with and without the excitation delayed by *t*, respectively. Moreover, Δ*E_t_*(ω)/*E*(ω) refers to the change value of the terahertz amplitude after the material is excited by pump light, which is obtained from Equation (1).

**Figure 3:**
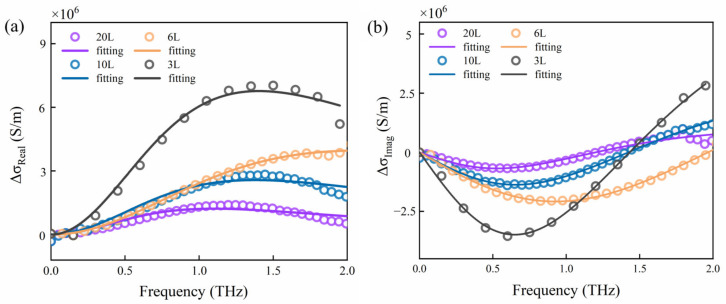
Changes in photo-induced conductivity Δσ of PtSe_2_ films with different layers. (**a**) Real part; (**b**) imaginary part.

Based on Equation (2), Figure 3 shows the real and imaginary part data values of the photo-induced conductivity change of PtSe_2_ films, where the hollow circles and solid line are the experimental and fitting curves, respectively, of the complex conductivity by the Drude–Smith (DS) model. It clearly shows that the peak values of the real part of the photo-induced conductivity change for 3L, 6L, 10L, and 20L PtSe_2_ films are about 7 × 10^6^ S/m, 4 × 10^6^ S/m, 3 × 10^6^ S/m, and 1.5 × 10^6^ S/m, respectively. One can obtain that the thinner the layers of the material, the higher the change value of the photo-induced conductivity, which means the surface photoconductivity of the material is better. The transient response to photo-excitation can be explained by the Drude–Smith model [42] and is described as

(3) $$\sigma_{DS}\left(\omega \right)=\frac{\varepsilon_{0}\omega_{p}{}^{2}\tau_{s}}{1-i\omega \tau_{s}}\left(1+\frac{c}{1-i\omega \tau_{s}}\right)$$

where *ε_0_, ω_p_, τ_s_,* and *c* represent the vacuum dielectric constant, plasma frequency, scattering time, and degree of localization and backscattering of carriers in the film, respectively. Based on the simulation data of the above equation in Figure 3, the Drude–Smith fitting parameters of the different layers of PtSe_2_ films are shown in Table 1. Furthermore, *ω_p_* is proportional to the concentration of the photocarrier of the material, indicating that the 3L PtSe_2_ film has a higher number of photogenerated carriers and better surface conductivity characteristics than other layers. The scattering time *τ_s_* is 70 fs, and the constant *c* is close to -1, indicating that the backscattering process is dominant in the PtSe_2_ film. Through experiments, it is found that the 3L PtSe_2_ film has larger conductivity change value Δ*σ_Real_* and larger plasma frequency *ω_p_*. Therefore, the 3L PtSe_2_ film has excellent surface photoconductivity and is more suitable for a terahertz modulator.

**Figure 4:**
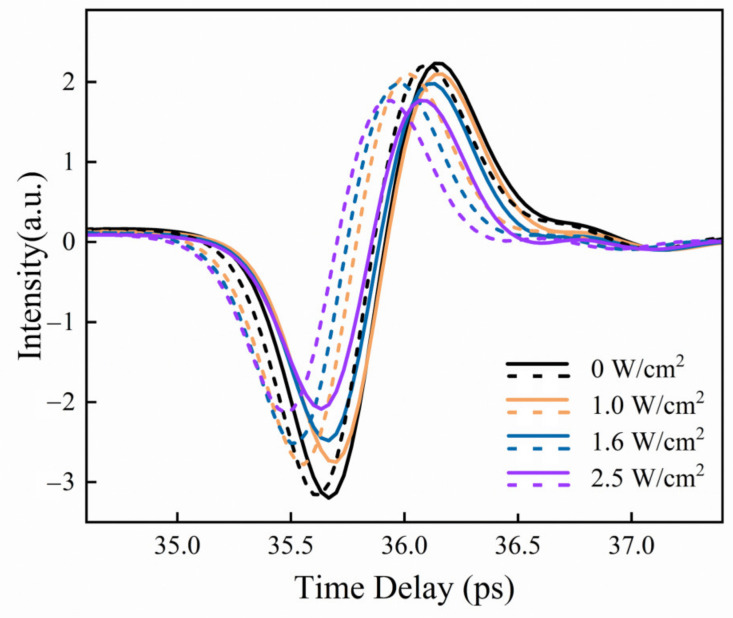
Time-domain transmission terahertz signals of PtSe_2_ devices at different pump power density, where solid and dashed lines represent 3L and 6L PtSe_2_ devices, respectively.

In addition, the terahertz modulation properties of 3L and 6L PtSe_2_ devices on high-resistance silicon substrates were also measured by THz-TDS. A continuous-wave laser with a wavelength of 1064 nm was used as the optical pump source. Figure 4 shows the transmission terahertz signals in 3L and 6L PtSe_2_ devices under a different pump power density, where the same color represents an identical pump condition for a different-layer PtSe_2_ device. Moreover, the solid and dashed lines represent 3L and 6L PtSe_2_ devices, respectively. The photogenerated carriers in PtSe_2_ devices are generated by pump light excitation, which can absorb terahertz waves and result in a reduction in amplitude in the time domain. With the increase in the incident laser power density from 0 W/cm^2^ to 2.5 W/cm^2^, the electric field amplitude of the sample shows a continuous declination, and the terahertz modulation effect can be clearly observed.

Modulation depth (*MD*), modulation bandwidth, and modulation rate are used to evaluate the performance of a terahertz modulator, where *MD* is defined as [40]

(4) $$MD=\left|\frac{T_{p}-T_{0}}{T_{0}}\right|$$

where *T*_p_ and *T*_0_ represent the transmittance of the sample in the corresponding frequency spectra after Fourier transform with and without optical pump, respectively.

**Figure 5:**
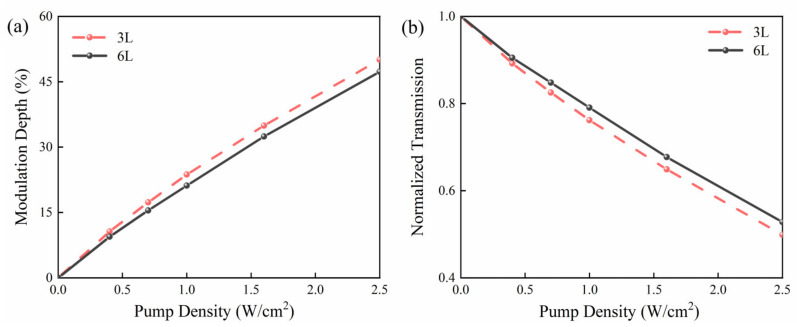
(**a**) Modulation depths of 3L and 6L PtSe_2_ devices at different pump power density. (**b**) Normalized transmittance signals at different pump power density of 3L and 6L PtSe_2_ devices.

Figure 5a shows the *MD* of 3L and 6L PtSe_2_ devices at different pump power density. The relationship between the normalized terahertz transmittance and pump power density for the two samples is shown in Figure 5b. The experimental results show that the modulation depth increases with the increase in the pump power density. At a pump power density of 2.5 W/cm^2^, the maximum *MD* of 3L and 6L PtSe_2_ devices can reach 50.9% and 47.1%, respectively. As shown in Figure 3, Figure 5 further verifies that the 3L PtSe_2_ device has better modulation effect. As the conductivity of the material increases, it will absorb more terahertz waves, which is shown as a decrease in the terahertz amplitude. After the device is excited, more photocarriers are generated in the high-resistance silicon due to the different thickness. Due to the charge gradient, the electrons generated in the silicon are transferred to the side of the PtSe_2_ films. The carrier mobility of PtSe_2_ is much higher than that of silicon, and its corresponding carrier relaxation time is shorter than that of silicon, which leads to a larger change in the conductivity of the modulator, thus achieving the effect of modulation enhancement.

## 4. Conclusions

The OPTP and TDS systems are used to measure the changes of surface photoconductivity, relaxation time, and modulation performance of PtSe_2_ devices with different layers. The material with fewer layers has better surface conductivity, and its change of the photo-induced conductivity is more helpful to change the modulation depth of the material than other layers. The relaxation times of 3L, 6L, 10L, and 20L PtSe_2_ films are 1.98 ± 0.14 ps, 2.28 ± 0.03 ps, 1.61 ± 0.02 ps, and 1.45 ± 0.02 ps, respectively, and the plasma frequencies *ω*_p_ fitted by the DS model are 0.23 THz, 0.21 THz, 0.17 THz, and 0.15 THz, respectively. For 3L PtSe_2_ devices, the MD can reach 50.9%. Our research work shows that few-layer PtSe_2_ is a more efficient terahertz modulation material.

## Figures and Tables

**Table 1:** Drude–Smith fitting parameters of different layers of PtSe_2_ films.

Parameters	*ω*_p_ (THz)	*τ_s_* (ps)	*c*
Layers			
3	0.23	0.07	−0.94
6	0.21	0.09	−0.96
10	0.17	0.11	−1
20	0.15	0.14	−1

## Data Availability

The data presented in this study are available on request from the corresponding author. The data are not publicly available due to restrictions.
